# Gambogic acid targets HSP90 to alleviate DSS-induced colitis via inhibiting the necroptosis of intestinal epithelial cells

**DOI:** 10.3389/fphar.2025.1586705

**Published:** 2025-05-19

**Authors:** Yuanyuan Wang, Siqi Liu, Keyi Lu, Erping Xu, Zhibin Wang

**Affiliations:** ^1^ Collaborative Innovation Center of Research and Development on the Whole Industry Chain of Yu-Yao, Henan Province, Henan University of Chinese Medicine, Zhengzhou, Henan, China; ^2^ Academy of Chinese Medical Sciences, Henan University of Chinese Medicine, Zhengzhou, Henan, China; ^3^ Department of Critical Care Medicine, School of Anesthesiology, Naval Medical University, Shanghai, China

**Keywords:** Gambogic acid, ulcerative colitis, necroptosis, Hsp90, IECs

## Abstract

Abnormal elevations in the mortality of intestinal epithelial cells (IECs) are indicative of intestinal inflammation. Necroptosis of IECs represents a pro-inflammatory form of cell death, and modulation of IECs necroptosis may mitigate subsequent intestinal inflammation and preserve the integrity of the intestinal barrier. Currently, safe and effective preventive measures are lacking. In the Traditional Chinese Medicine theory, necroptosis of IECs leads to the destruction of the intestinal barrier in a manner associated with “heat and toxicity”, exacerbating intestinal inflammation. Heat shock protein 90 (HSP90) has been identified as a regulator of key proteins involved in necroptosis signal pathway including RIPK1/3 and MLKL. Gambogic acid (GA), the primary active compound found in *Garcinia hanburii* Hook. f., a traditional Chinese medicine used for detoxification and hemostasis, has not been studied for its potential therapeutic effects in ulcerative colitis previously. This study investigated the protective effects of GA on dextran sodium sulfate (DSS)-induced colitis in mice, as well as the underlying molecular mechanisms. GA was observed to significantly ameliorate DSS-induced enteritis and enhance intestinal barrier function. Concurrently, it reduced the phosphorylated expression levels of RIPK1/3 and MLKL. The underlying mechanism may be related to the suppression of HSP90 expression.

## 1 Introduction

Ulcerative colitis (UC) is a chronic, idiopathic inflammatory disease of the colon and rectum characterized by intestinal inflammation and barrier damage, clinically presenting symptoms such as diarrhea, mucous bloody stools, abdominal pain, and urgency (Le Berre et al., 2023). The incidence of UC is rising globally due to alterations in lifestyle and dietary habits, thereby presenting a significant threat to public health (Lopes et al., 2022). Statistics show that up to 25%–30% of UC patients require colon resection surgery, and over 20% of inflammatory bowel disease patients will develop colorectal cancer within 30 years. UC is classified by the World Health Organization as a “modern refractory disease” (Eisenstein, 2018; Ng et al., 2017). Presently, the primary pharmacological treatments for UC encompass amino salicylates, corticosteroids, and immune-suppressants, which are used to treat symptoms by suppressing inflammation. However, these treatments are associated with significant challenges, including high levels of drug resistance, numerous adverse reactions, and an elevated risk of tumor development (Kucharzik et al., 2020). UC has become a pressing social public health issue, therefore the discovery of new effective drugs for prevention and treatment is urgent required.

Epithelial damage and increased intestinal permeability in IECs are the known characteristics of inflammatory bowel disease, and an increasing volume of research is being undertaken to investigate this as a potential therapeutic target (Li et al., 2024; Wang Y. et al., 2024; Yang et al., 2024). The abnormal death of IECs is the most direct cause of IEC damage and subsequently leads to further impairment of the tight junction (TJ) barrier (Patankar and Becker, 2020). Factors such as inflammation, radiation, and pharmacological agents can induce abnormal IEC death, thereby compromising tight junctions between cells and increasing intestinal permeability. Harmful substances subsequently infiltrate the bloodstream via the intestinal wall, thereby compromising barrier integrity. In contrast, aberrant death of IECs results in the release of inflammatory mediators, which amplify the level of intestinal mucosal inflammation and further deteriorate barrier function. Inflammation is widely recognized as the principal etiological factor in UC, with abnormal IECs death playing a pivotal role in the disruption of intestinal barrier function characteristic of this condition.

Necroptosis is inflammatory cell death that links inflammatory response which differs from apoptosis and traditional necrosis. The process of necroptosis is marked by organelle swelling, loss of membrane integrity, and the release of cytoplasmic contents. Necroptosis represents a critical programmed cell death pathway in IECs induced by UC (Patankar and Becker, 2020). Tumor necrosis factor-α (TNF-α), a multifunctional pro-inflammatory cytokine, is considered the primary driving force behind the death of IECs. TNF-α is a principal inducer of necroptosis, a cell death pathway that has been extensively investigated to date (Kalliolias and Ivashkiv, 2016).

Upon TNF-α stimulation, TNF receptor 1 (TNFR1) recruits various proteins to assemble a complex at the plasma membrane, facilitating nuclear factor kappa-light-chain-enhancer of activated B cells (NF-κB) signaling. In conditions when Caspase-8 activity is inhibited, receptor-interacting protein kinase 1 (RIPK1) and receptor-interacting protein kinase 3 (RIPK3) interact to form a necrosome, subsequently activating mixed lineage kinase domain-like kinase (MLKL) (Oberst et al., 2011). MLKL then oligomerized and translocated to the cell membrane, culminating in membrane rupture and the execution of necroptosis. Upon rupture of the cell membrane, a substantial release of intracellular contents occurs, which subsequently activates pattern recognition receptors and induces severe intestinal inflammation. This dysregulated immune response, coupled with necroptosis, establishes a vicious cycle that perpetuates intestinal inflammation (Pasparakis and Vandenabeele, 2015). Furthermore, the excessive loss of IECs compromises the integrity of tight junctions, thereby increasing intestinal permeability and impairing the overall function of the intestinal barrier.

Clinical data indicate that, within the inflammatory tissues of patients with inflammatory bowel disease, there is a significant reduction in both the number of IECs and the number of dead cells in the crypt basal area. Concurrently, there is an observed increase in the levels of RIPK3 and MLKL, alongside a decrease in Caspase-8 levels (Gunther et al., 2011; Pierdomenico et al., 2014). Moreover, RIPK3-mediated necroptosis significantly decreases the levels of tight junction proteins like ZO-1, Cadherin E, and Occludin thus weakening intestinal mucosa permeability (Negroni et al., 2017). These findings substantiate the notion that necroptosis of IECs is a critical pathogenic mechanism contributing to the disruption of the epithelial barrier and the subsequent onset of intestinal inflammation. The inhibition of the RIPK3 or the application of necroptosis inhibitors (Lee et al., 2020; Wang Y. et al., 2024; Zhang et al., 2020) has been shown to mitigate necroptosis in IECs and ameliorate damage to the intestinal barrier, thereby alleviating UC. Consequently, the development of therapeutic agents targeting the inhibition of necroptosis in IECs holds significant promise for UC treatment. Such interventions could directly diminish epithelial cell loss, thereby enhancing intestinal barrier function, while concurrently reducing intestinal inflammation and further improving intestinal permeability.

In TCM theories, “inflammation” is regarded as a significant pathological outcome of the conflict between Evil Qi (pathogenic factors) and Zheng Qi (the body’s vital energy), akin to the concept of “heat and toxicity”. The “heat and toxicity” is one of the pathogenesis of intestinal erosion ulcers, as delineated by Xichun Zhang in “Yixue zhongzhong canxilu”. The description, “Heat and toxicity invades the skin in the intestine, persisting until it decays, and it also causes the skin to ulcerate,” aligns with the contemporary understanding of the pathogenesis of UC during its acute stage (Zhang, 2016). Therefore, the inhibition of excessive necroptosis in IECs to control inflammation consistent with the TCM principle of “clearing heat poison to dispel evil” (Zhou, 2013). In this study, we established a self-established compound library of traditional Chinese medicines possessing the function of clearing heat, detoxifying, and reducing swelling, and identified Gambogic acid (GA) from Garcinia hanburyi Hook. f. having anti-necroptosis activity (Supplementary Figure S1). However, the role and mechanism of GA in UC remain undocumented.

Therefore, this study examined the impact of GA on the treatment of UC and investigated the underlying mechanisms by which GA inhibits necroptosis. This exploration was conducted through bioinformatics analyses and substantiated by molecular docking, molecular dynamics simulations, and both *in vitro* and *in vivo* experimental validation methodologies.

## 2 Materials and methods

### 2.1 Reagents

We obtained dextran sulfate sodium (DSS) (MW: 36,000–50,000, cat#CD4421) from Coolaber (Beijing, China). GA was acquired from TargetMol (T6185), and the purity is 98.38% (Lot:150301). Necrostain-1 (Nec-1) (S8037) was acquired from Selleck. We purchased Human-TNF-alpha (C008) from Novoprotein, SM-164 Hydrochloride (HY-15989A) from Med Chem Express, and z-VAD-fmk (T6013) from TargetMol. Cell Signaling Technology supplied anti-RIPK1 (3493S), anti-HSP70 (4872), anti-EGFR (4267), and human-specific anti-phospho-RIPK1 (65746S). We obtained mouse-specific anti-phospho-RIPK1 (BX60008) from Biolynx. Abcam provided anti-RIPK3/p-RIPK3 (ab209384) and mouse-specific anti-phospho-RIPK3 (ab195117). We obtained the RIPK3 polyclonal antibody (17563-1-A) and Anti-MLKL (66675-1-Ig) from Proteintech. Anti-MLKL (ab184718) and human-specific anti-phospho-MLKL (ab187091) were sourced from Abcam, while mouse-specific anti-phospho-MLKL (bsm-54104R) came from Bioss. Servicebio supplied the HSP90 (GB12284), HSP70 (GB12244), Occludin (GB111401), and ZO-1 (GB111402) antibodies. The Anti-GAPDH (ab181602) was purchased from Abcam, and the BCA protein assay kit (CW0014S) was acquired from CWBIO. We obtained the HRP Goat anti-rabbit IgG (abs20040) from Absin and the high-intensity ECL Western blotting substrate from Tanon.

### 2.2 Cell culture

The HT-29 cell line was procured from Procell Life Science and Technology Co., Ltd. (catalog number CL-0118; Wuhan, China). The cells were cultured in growth medium (Procell, CM-0118) supplemented with 10% fetal bovine serum (BI, C04001-500) and 100 U/mL penicillin/streptomycin (Gibco, 15140–122) under conditions of 37°C and 5% CO_2_.

### 2.3 Anti-necroptosis activity of GA

HT-29 cells were seeded in 96-well plates at 10,000 cells per well, with triplicates for each group and cultured for 12 h. GA was diluted in a culture medium supplemented with 10 nM SM-164 and 20 μM Z-VAD-FMK to achieve final concentrations ranging from 5 μM to 0.08 μM. Following 12 h of cell culture, the culture medium was replaced with the prepared GA solutions. After a 30-min pre-treatment with GA, the cells were stimulated with 20 ng/mL recombinant human TNF-α. This marked the initiation of the TNF-α-SMAC-z-VAD-FMK (TSZ) treatment, which involved the administration of human TNF-α in the presence of SM-164 and Z-VAD-FMK as necroptosis inducers. Cell viability was assessed after 12 h using the CellTiter-Lumi II Luminescent Cell Viability Assay Kit (catalog number C0056S; Beyotime Institute of Biotechnology).

### 2.4 Double staining of live/dead cells

HT-29 cells were harvested as previously outlined, and 100 μL of a Calcein-AM/PI working solution (dilution 1:1,000) was subsequently added to each well following a 12-h incubation period. The plates were then incubated for an additional 30 min at 37°C, shielded from light exposure. Cellular viability in each group was assessed using a microscope (Nikon, Tokyo, Japan), where red fluorescence indicated non-viable cells and green fluorescence indicated viable cells.

### 2.5 Animals

The animal experiments were approved by the Animal Care and Use Committee of Henan University of Chinese Medicine (IACUC-S202403126). Male C57BL/6J mice aged 6–7 weeks were obtained from Liaoning Changsheng Biological Co., Ltd in Liaoning, China. The mice were housed in controlled conditions with regulated temperature and humidity, a 12-h dark-light cycle, and provided with standard dry diet and tap water *ad libitum*.

### 2.6 UC model induced by DSS

A murine model of DSS-induced UC was utilized in this study. After a 1-week acclimation period on a standard diet, the mice were randomly allocated into two groups: a control group (Con) and a DSS. The control group was provided with standard chow and water *ad libitum*, whereas the DSS group received 3.0% DSS in their drinking water for 7 days to induce UC. Following this induction period, the DSS group was further subdivided into four treatment cohorts: DSS-only, high-dose GA (GA 5 mg/kg), low-dose GA (GA 0.5 mg/kg) and Nec-1 (5 mg/kg), with each cohort comprising six mice. The dosages of GA were determined based on prior research and preliminary experiments (Thongrung et al., 2025; Yu et al., 2019). The DSS-only group continued on a standard chow diet with water. The treatment groups received their respective interventions once daily for seven consecutive days, with GA 5 mg/kg and GA 0.5 mg/kg administered orally via gavage, and Nec-1 delivered intraperitoneally once a day. On the 14th day of the study, all animals were euthanized by certified personnel through the administration of an overdose of sodium pentobarbital (150 mg/kg, intraperitoneally). Following the induction of unconsciousness, cervical dislocation was performed as a secondary measure to ensure the complete cessation of cerebral activity. The death of each animal was confirmed by the absence of detectable cardiac activity and the loss of corneal reflexes. All animal procedures were conducted at the Animal Experimental Center of Henan University of Chinese Medicine. Blood and colonic tissue samples were collected post-mortem. The length of the colon was measured, and distal of colon tissues were prepared for histological analysis by embedding in paraffin. Tissues were initially fixed in 4% paraformaldehyde for 24 h at room temperature, followed by dehydration in a graded alcohol series. Subsequently, the tissues were immersed in wax and embedded using an embedding machine. Following dehydration, the tissues were immersed in paraffin wax and subsequently embedded utilizing an embedding apparatus. The wax blocks were then cooled at −20°C, trimmed, and sectioned to a thickness of 4 μm.

### 2.7 Disease activity index (DAI)

The DAI score combines three parameters: body weight loss, blood in stool, and diarrhea severity. Body weight loss is scored from 0 (none) to 4 (>20%). Blood in stool is scored from 0 (none) to 4 (gross bleeding). Diarrhea severity was scored as follows: 0 (normal), 1 (soft), 2 (soft and pasty), 3 (soft and pasty adhering to the anus), and 4 (liquid). The final DAI score was the sum of these scores (Wang Y et al., 2024; Yan et al., 2018; Zhang et al., 2023; Zhong et al., 2023).

### 2.8 FITC-dextran assay

After a 4-h fasting period, mice were orally gavaged with 600 mg/kg of FITC-dextran (Sigma, FD4, molecular weight: 3,000–5,000) 4 h before euthanasia. Blood samples were obtained and centrifuged, followed by dilution of the resulting supernatant with PBS at a 1:5 ratios. The levels of FITC-dextran in the serum were then measured using the SpectraMax M5 fluorescence assay with excitation at 488 nm and emission at 525 nm.

### 2.9 HE and PAS staining

The colon, heart, liver, and kidney tissues of mice were fixed using 4% paraformaldehyde, embedded in paraffin, sectioned, and stained with hematoxylin and eosin. Additionally, the colon tissue was stained with periodic acid-Schiff (PAS) following the manufacturer’s instructions.

### 2.10 ELISA

Blood samples of mice were centrifuged 1,000 g for 15 min and supernatant was taken. Serum release of TNF-α was measured using an enzyme-linked immunosorbent assay kit (ELISA) according to manufacturer’s instructions (Elabscience, E-EL-M3063).

### 2.11 Immunofluorescence

Paraffin-embedded intestinal tissue sections were cut into slices. After removing the paraffin, the epithelial monolayer was immunostained. Slides were incubated overnight with antibodies at 4°C, rinsed with PBS four times, and then incubated with a secondary antibody for 1 h in the dark. They were stained with DAPI (G1012, Servicebio, China) for 10 min at room temperature, rinsed with PBS, and treated with an anti-fluorescence quencher. Following the primary antibodies, AffiniPure goat anti-rat IgG (1:200) (GB21303, Servicebio, China) and goat anti-mouse IgG (1:200) (GB22301, Servicebio, China) were used as secondary antibodies. The fluorescent images of samples were captured using confocal microscopy.

### 2.12 Western blot

HT-29 cells were cultured in 6-well plates at a density of 100,000 cells per well and treated with varying concentrations of GA or stimulated with TSZ for different durations to assess their anti-necroptosis activity. Following treatment, the cells were lysed in RIPA Lysis Buffer (CWBIO, Jiangsu, China) and the protein concentration was determined using a BCA protein assay kit (CWBIO, Jiangsu, China). Subsequently, the protein samples were separated by 10% SDS-PAGE and transferred onto PVDF membranes (Millipore, Bedford, MA, United States). Subsequently, the membranes were treated with 5% BSA for a duration of 2 h, followed by an overnight incubation at 4 °C with different primary antibodies at a 1:1,000 dilution. After a 1-h incubation with HRP-conjugated secondary antibodies, the membranes were scanned using an imaging system (Bio-Rad, Hercules, United States) and the optical density of the bands was analyzed using ImageJ software.

### 2.13 KINOMEscan™ analysis

GA’s binding affinities for RIPK1, RIPK3, and MLKL kinases were measured using KINOMEscan™ assay by DiscoveRx Corporation following the protocol provided by the service provider. In brief, the RIPK1, RIPK3, and MLKL kinases were labeled with DNA for qPCR detection. The liganded beads were saturated with biotin and washed with blocking buffer to remove unbound ligand and reduce nonspecific binding. Binding reactions were then set up in binding buffer with kinases, liganded affinities, and test compounds. GA was prepared at a concentration of 5 mM in DMSO. The dissociation constants (Kds) were determined utilizing an 11-point, 3-fold serial dilution of the compound, accompanied by three control points containing DMSO. GA was subsequently diluted into the assays, resulting in a final DMSO concentration of 0.9%, and the assays were conducted in polypropylene 384-well plates. The reactions were conducted in a volume of 0.02 mL and incubated at room temperature with agitation for a duration of 1 hour. Subsequently, the beads were washed using a wash buffer and re-suspended in an elution buffer containing a non-biotinylated ligand, followed by an incubation period of 30 min. The concentration of kinase in the eluates was quantified using quantitative PCR (qPCR).

### 2.14 Network construction and analysis

Targets related to UC and necroptosis, as well as potential targets of GA, were identified using the GeneCards database (https://www.genecards.org/). The intersection of genes associated with UC, necroptosis, and GA was subsequently utilized to perform a network analysis of PPI via the STRING database (https://cn.string-db.org/). This analysis aimed to identify potential therapeutic targets of GA in the treatment of UC through the inhibition of necroptosis.

### 2.15 Molecular docking

The structure of HSP90α was retrieved from the Protein Data Bank (PDB ID: 8AGL), and the three-dimensional structure of Gambogic acid was obtained from the National Center for Biotechnology Information (NCBI) database (PubChem CID: 9852185). The interaction between HSP90α and Gambogic acid was analyzed using AutoDock Vina software, followed by visualization of the resulting complex using Maestro 2021 software.

### 2.16 Molecular dynamics (MD) simulation

Based on molecular docking studies, the Desmond program was employed to simulate the molecular dynamics of the aforementioned small molecule complexes. The OPLS2005 force field was utilized to parameterize both proteins and small molecules, while the TIP3P model was applied to represent solvated water. The protein-small molecule complex was positioned within a cubic water box and solvated, followed by neutralization of the system’s charge through the addition of 0.150 M chloride and sodium ions. Initially, the system’s energy is minimized through 50,000 steps employing the steepest descent minimization method. Subsequently, the positions of the heavy atoms are constrained to facilitate the execution of NVT (constant number of particles, volume, and temperature) and NPT (constant number of particles, pressure, and temperature) equilibration, each conducted over 50,000 steps. During these equilibration phases, the system’s temperature is maintained at 300 K, and the pressure is held at 1 bar. Following the completion of these equilibration stages, an unrestricted simulation is performed for 100 nanoseconds. Throughout this simulation, energy and coordinate data are recorded every 10 picoseconds. Post-simulation, Maestro 2021 software is utilized to generate interactive plots and dynamic trajectory animations.

### 2.17 Statistical analysis

The one-way analysis of variance followed by Tukey’s multiple comparision test was employed to assess variations among groups as indicated by the mean values ±standard deviation. A significance level of *P* < 0.05 was deemed statistically significant.

## 3 Results

### 3.1 GA identified as a potential necroptosis inhibitor

**FIGURE 1:**
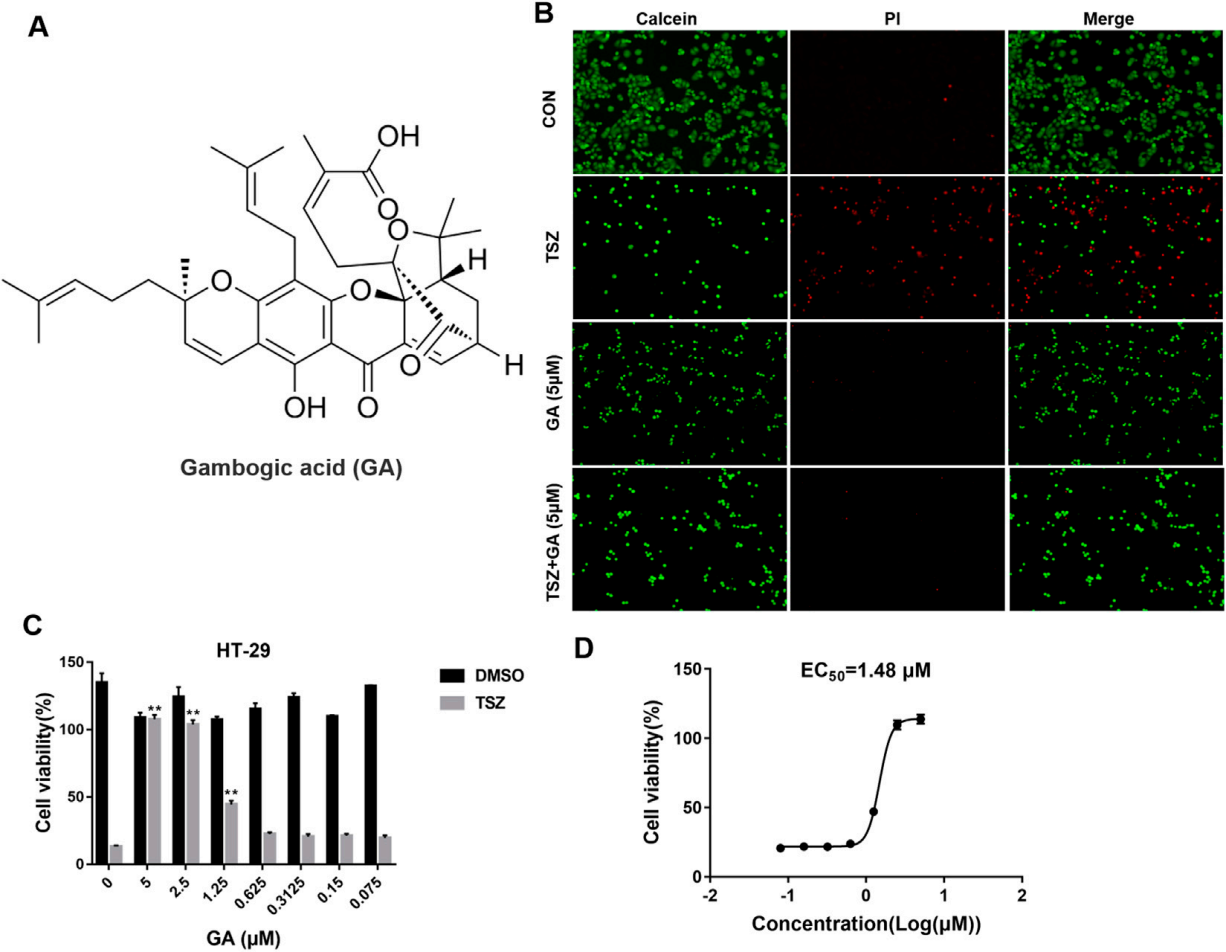
GA as a potential necroptosis inhibitor. **(A)** Chemical structure of GA; **(B)** Representative images of calcein-AM/PI staining of GA subjected to various treatments for a duration of 12 h; **(C)** HT-29 cells were treated with a specified concentration of GA, followed by stimulation with TSZ for 12 h, and cell viability was assessed using the CellTiter-Glo chemiluminescence assay; **(D)** Half-maximal effective concentration (EC_50_) of GA for the inhibition of necroptosis. **P < 0.01 compared to the TSZ group.

Traditional Chinese medicine represents a valuable repository for the discovery of novel pharmacological agents. In this study, a comprehensive library of Chinese medicinal compounds was developed utilizing a commercial Chinese medicine monomer library (HY-L065, MCE). This extensive collection includes compounds known for their antipyretic, detoxifying, and anti-inflammatory properties, featuring 24 derivatives sourced from Taraxasterol, Arenobufagin, Gastrodin, etc. Within the established HT-29 cell necroptosis model induced by TNF-α, SM-164, and Z-VAD-FMK (TSZ) (Liu et al., 2024), GA was identified as a potent inhibitor of necroptosis (Supplementary Figure S1). Figure 1A illustrates the structural formula of GA. We further investigated the anti-necroptosis activity of GA *in vitro* using a TSZ-induced necroptosis model of HT-29 cells. Various concentrations of GA were incubated with the cells for 12 h. After GA treatment, more viable cells were observed, as shown by the Calcein/PI Cell Viability/Cytotoxicity Assay Kit (Figure 1B). Calcein AM stained live cells green, while Propidium Iodide marked dead cells red. Moreover, GA significantly enhanced cell viability, with an EC_50_ value of 1.48 μM (Figures 1C,D).

### 3.2 GA inhibits HT-29 cell necroptosis signaling

**FIGURE 2:**
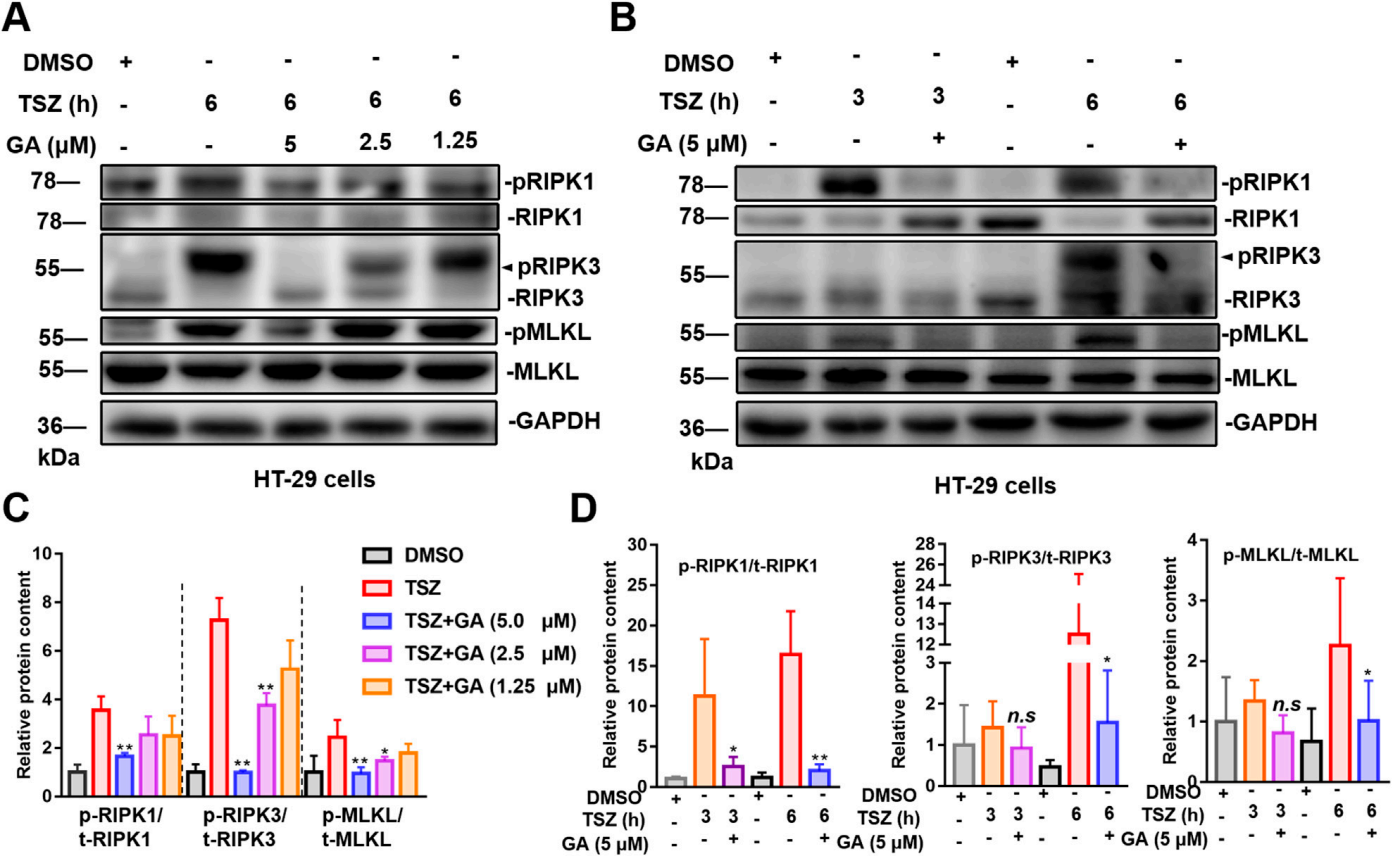
GA inhibits the activation of the necroptosis signaling pathway. **(A)** HT-29 cells were exposed to varying concentrations of GA for 6 h **(B)** HT-29 cells were pretreated with GA (5 µM) for 30 min, followed by treatment with TSZ for specified durations. **(C)** The quantitative analysis of the grayscale intensities of protein bands with different concentrations of GA for 6 h. **(D)** The quantitative analysis of the grayscale intensities of protein bands with different time. Data are expressed as the mean ± standard deviation (n = 3).

RIPK1, RIPK3, and MLKL are critical proteins involved in the necroptosis pathway (Wang M. et al., 2024). The phosphorylation of RIPK1 and RIPK3 leads to the formation of the necrosome, which subsequently induces the phosphorylation and oligomerization of MLKL, ultimately resulting in cell membrane rupture. Consequently, we assessed the phosphorylation status of RIPK1, RIPK3, and MLKL in the presence of GA. Experimental results demonstrated that GA inhibited the phosphorylation of RIPK1, RIPK3, and MLKL (Figures 2A,C). GA could inhibit the phosphorylation status of RIPK1, RIPK3, and MLKL even when TSZ continuously stimulated and enhanced the activation of necroptosis pathway (Figures 2B,D).

### 3.3 GA alleviates DSS-induced UC injury in mice

**FIGURE 3:**
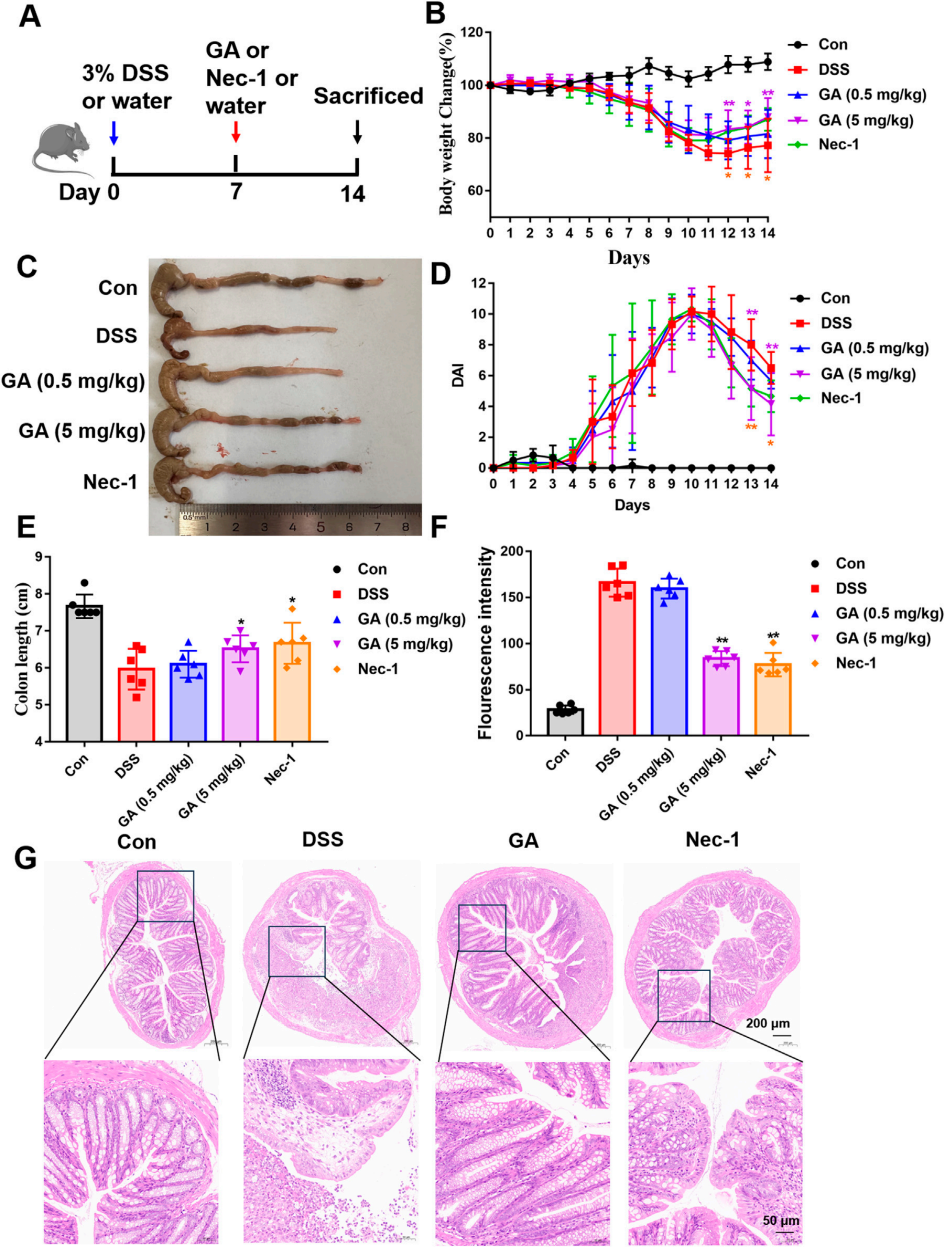
Protective effects of GA on DSS-induced UC in mice. **(A)** Schematic representation of the DSS-induced mouse model of UC and the administration protocol; **(B)** Variations in body weight of mice throughout the experimental period; **(C)** Representative images of mouse colons; **(D)** DAI scores during the experiment; **(E)** Colon length on day 14; **(F)** Serum FITC-glucan fluorescence intensity; **(G)** Hematoxylin and Eosin (HE) staining of representative colon tissue sections. *P < 0.05 and **P < 0.01 compared with the DSS group. The one-way analysis of variance followed by Tukey’s multiple comparision test was employed to assess variations among groups as indicated by the mean values ±standard deviation. A significance level of P < 0.05 was deemed statistically significant.

To ascertain the protective effects of GA against UC, a DSS-induced mouse model was employed. The schematic diagram is depicted in Figure 3A. Nec-1, a well-documented inhibitor of necroptosis, was utilized as a positive control (5 mg/kg). GA was administered at dosages of 0.5 mg/kg (low dose) and 5 mg/kg (high dose). The experimental results indicated that GA markedly alleviated the symptoms associated with DSS-induced UC. Compared to the model group, the GA (5 mg/kg) group exhibited significant weight recovery (Figures 3B,C), a reduced Disease Activity Index (DAI) score (Figure 3D), improved stool blood and quality, and increased colon length (Figure 3E). Additionally, the FITC fluorescence intensity indicated a decrease in intestinal permeability (Figure 3F). The therapeutic effects observed in the GA (5 mg/kg) group were comparable to those in the Nec-1 group. As the body weight and DAI scores was most significantly improved in the high-dose group, the GA (5 mg/kg) was selected in subsequent experiments. In order to evaluate the degree of intestinal injury, the pathological status was determined by HE staining. The results indicated that GA reduced submucosal edema in the colon, and ameliorated inflammatory infiltration (Figure 3G).

### 3.4 GA alleviates intestinal inflammation and intestinal barrier function in UC mice

The extended duration of UC and sustained chronic inflammation can result in the development of mucosal polyps, the shallowing or disappearance of colonic pockets, and tubular alterations in the intestinal wall (Pai et al., 2018; Peyrin-Biroulet et al., 2015). These changes may also lead to dysfunction or loss of the mucosal barrier and peristalsis. Despite the complete alleviation of clinical symptoms in some UC patients, mucosal inflammation may persist. Currently, the most widely accepted therapeutic objective among both domestic and international scholars is the healing of mucosal tissue. This approach aims to prolong remission periods, reduce recurrence rates, and diminish the risk of cancer (An et al., 2022; Chelakkot et al., 2018).

Among the intercellular connections within the intestinal mucosa, the tight junction (TJ) at the apical region of the mucosal epithelium is a critical structure that regulates cellular permeability and impedes the infiltration of deleterious substances, including macromolecular microorganisms and metabolites. This junction is primarily constituted of transmembrane proteins such as Claudin, Occludin, and Junctional Adhesion Molecules (JAMs), as well as junctional complex proteins (e.g., ZO-1, ZO-2) and elements of the cytoskeleton. These components collectively play a pivotal role in modulating the epithelial barrier function and maintaining cellular permeability (Capaldo et al., 2017).

**FIGURE 4:**
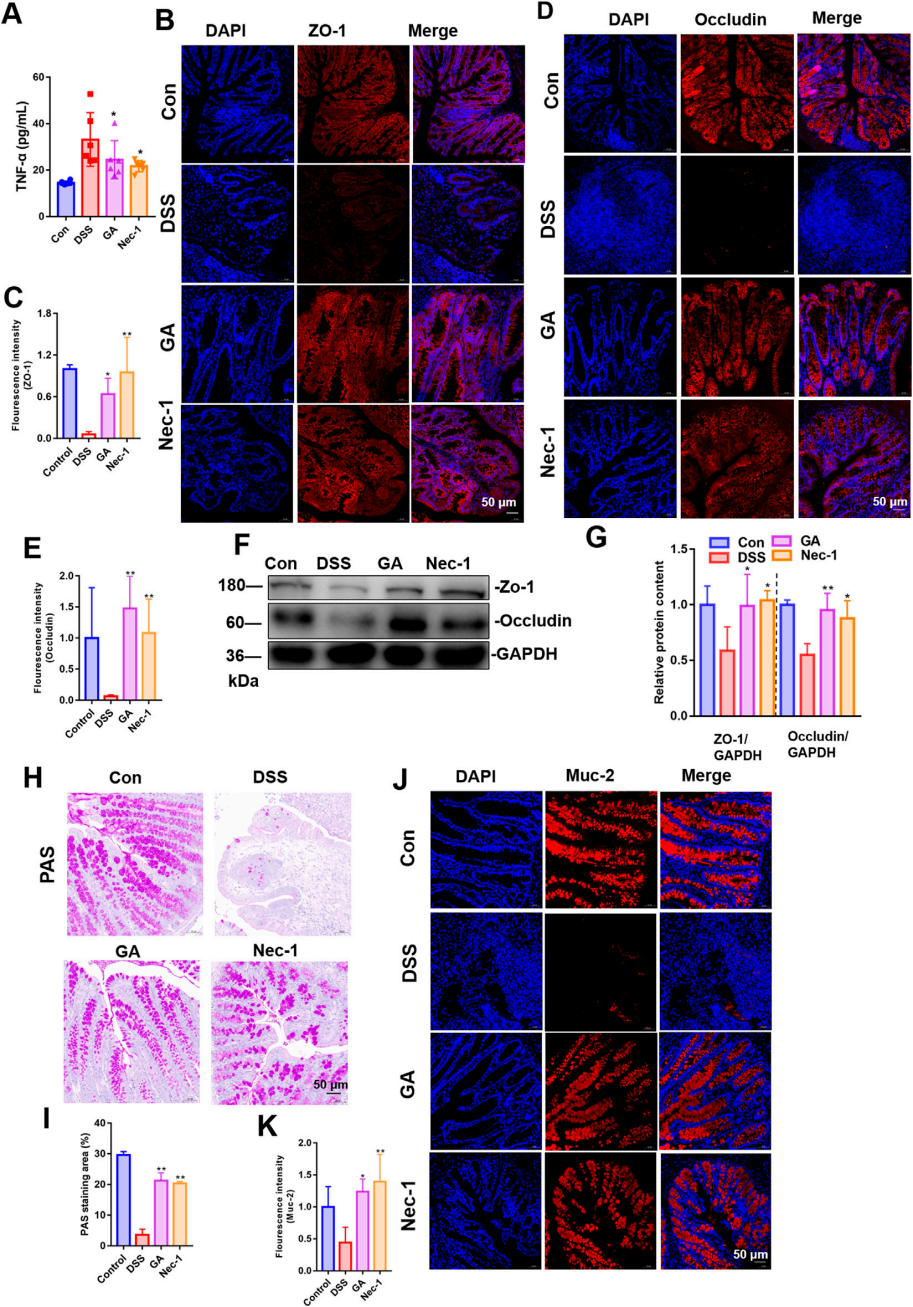
Impact of GA on intestinal inflammation and barrier function in UC mice. Immunofluorescence was used to assess GA’s effect on ZO-1 **(A)** and Occludin **(B)** distribution and expression in DSS-induced UC mice. **(C)** Representative PAS staining images of colon tissue; **(D)** Quantitative analysis of fluorescence intensity of ZO-1. **(E)** Quantitative analysis of fluorescence intensity of Occludin. **(F)** Quantitative analysis of PAS staining area. **(G)** Quantitative analysis of fluorescence intensity of Muc-2. **(H)** The distribution and expression levels of Muc-2 in colon tissues were assessed using immunofluorescence techniques. **(J)** Quantitative analysis of fluorescence intensity of ZO-1 and Occludin in mice colon tissue. **(I)** Representative images of immunoblotting for ZO-1 and Occludin (n = 3). **(J)** The quantitative analysis of the grayscale intensities of protein bandsin in colon tissues. **(K)** ELISA measured GA’s impact on serum TNF-α levels in these mice. **P < 0.01 vs. DSS group.

To assess the impact of GA on intestinal barrier function and inflammation, we measured serum TNF-α levels and examined the distribution and expression of Zo-1 and Occludin-1 in the intestinal tissue. The experimental results demonstrated that GA significantly ameliorated intestinal inflammation and barrier dysfunction induced by DSS, reduced serum levels of the inflammatory factor TNF-α (Figure 4A) and enhanced the expression of ZO-1 (Figures 4B,C) and Occludin-1 (Figures 4D,E) in colonic tissue. Western blot results further suggested that ZO-1 and Occludin-1 expression was increased significantly in GA groups (Figures 4F,G).

The intestinal mucosal barrier’s mechanical component includes IECs, their secreted mucus, and intercellular junctions (Macdonald and Monteleone, 2005). The mucus, antimicrobial peptides, and Muc-2 mucin produced by IECs are crucial for defending against bacterial and macromolecular invasion, maintaining the barrier’s normal function (Allaire et al., 2018; Delgado et al., 2016).

We employed PAS staining to quantify the intestinal goblet cells, assessed the expression and localization of mucin protein MUC-2 to determine the population of IECs. The quantity of goblet cells was assessed using PAS staining, while the distribution and expression levels of Muc-2 in colon tissues were evaluated through immunofluorescence techniques. The findings indicated that, in comparison to the DSS model group, GA treatment resulted in an increased number of goblet cells (Figures 4H,I), and an elevated expression of Muc-2 (Figures 4J,K).

### 3.5 GA alleviates the necroptosis of villi IECs in UC mice

**FIGURE 5:**
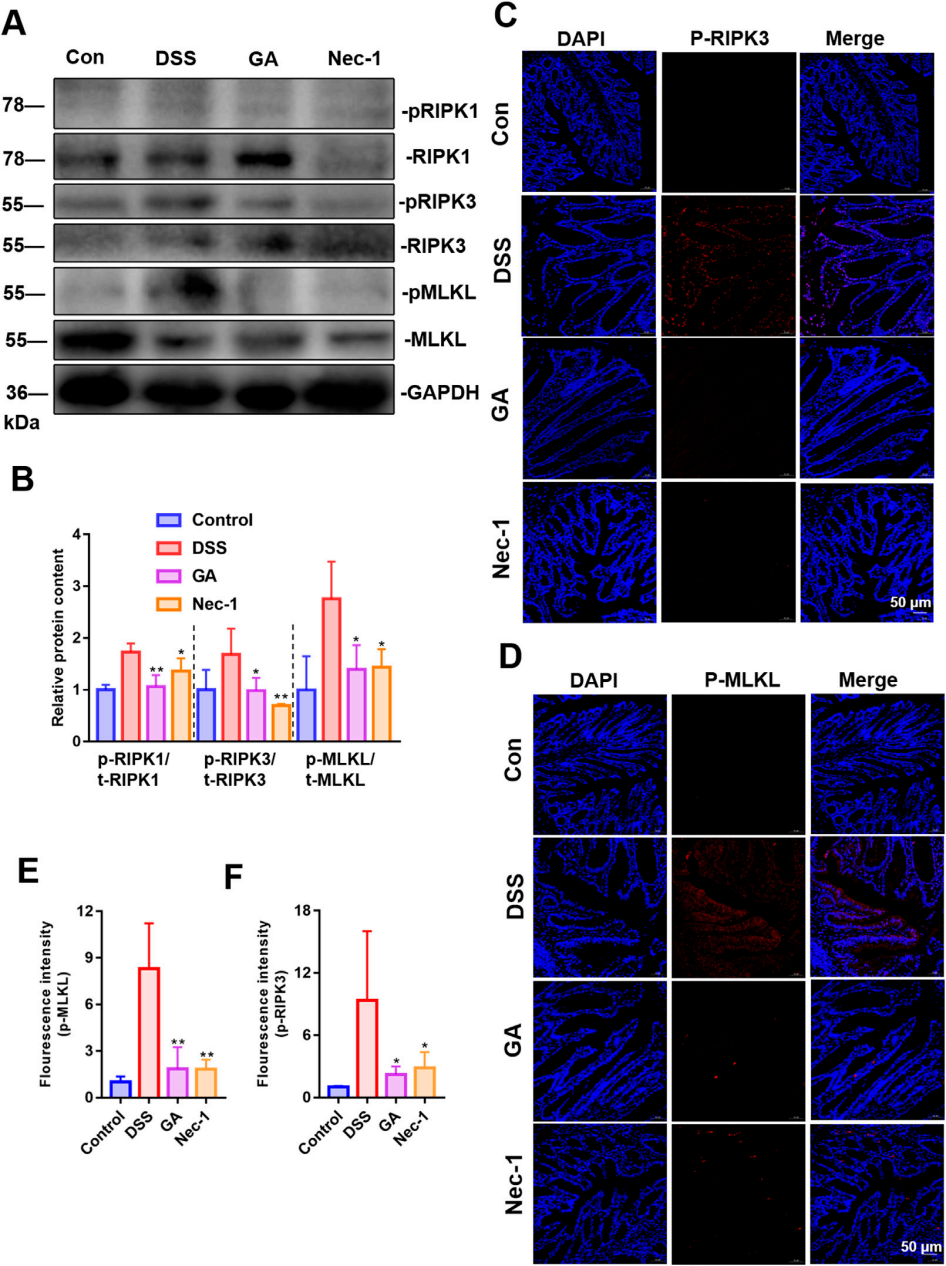
Effect of GA on necroptosis of villi IECs in UC mice. **(A)** Representative images of immunoblotting for RIPK1/3 and MLKL (n = 3). **(B)** The quantitative analysis of the grayscale intensities of protein bandsin in colon tissues. **(C)** The distribution and expression levels of p-RIPK3 in colon tissues. **(D)** The distribution and expression levels of p-MLKL in colon tissues. **(E)** Quantitative analysis of fluorescence intensity of p-MLKL. **(F)** Quantitative analysis of fluorescence intensity of p-RIPK3.

Abnormal necroptosis of IECs induces and exacerbates intestinal inflammation. The increased phosphorylation of RIPK1, RIPK3, and MLKL constitutes a crucial pathological process in necroptosis (Wang M et al., 2024). To evaluate the impact of GA on the necroptosis of villi IECs, we assessed the phosphorylation levels of RIPK1, RIPK3, and MLKL (Figures 5A,B), as well as the distribution of phosphorylated RIPK3 and MLKL within IECs (Figures 5C–F). Our findings indicate that GA can inhibit the necroptosis of villi IECs in UC mice, thereby enhancing intestinal barrier function and mitigating intestinal inflammation.

### 3.6 GA had no direct kinase inhibition function on RIPK1, RIPK3 or MLKL

**FIGURE 6:**
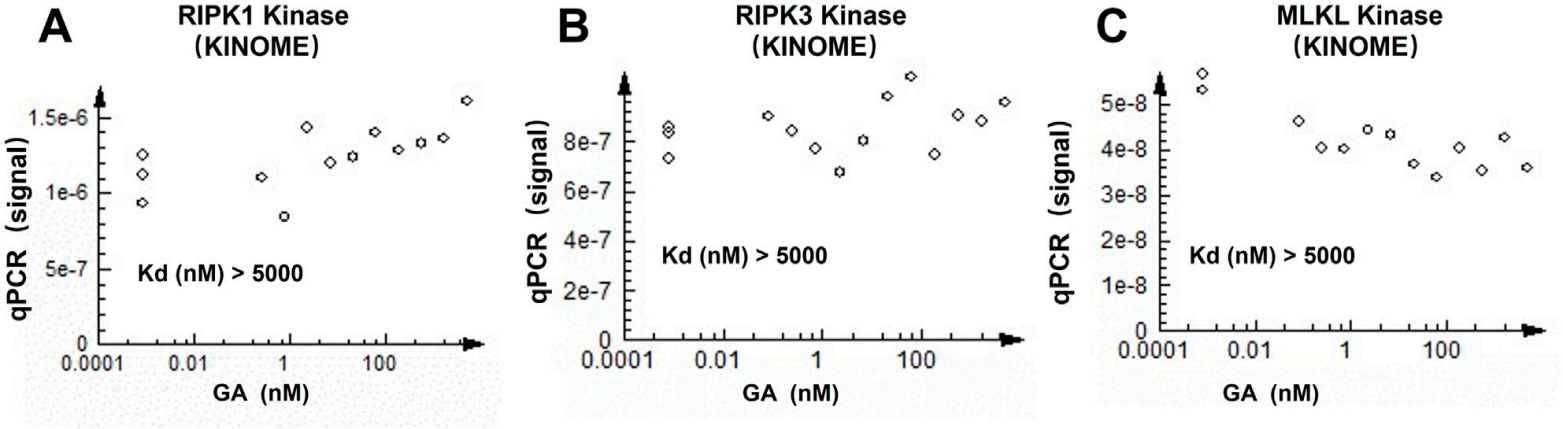
Effect of GA on the kinases activity of RIPK1, RIPK3 and MLKL. Effect of GA on the kinases activity of RIPK1 **(A)**, RIPK3 **(B)** and MLKL **(C)**.

Given that GA has been demonstrated to inhibit the phosphorylation levels of RIPK1, RIPK3, and MLKL both *in vivo* and *in vitro*, it is imperative to ascertain whether GA directly inhibits the kinase activity of RIPK1, RIPK3, or MLKL. To this end, the KINOMEscan™ assay was employed. The results indicated that GA does not inhibit the ligand binding of RIPK1 (Figure 6A), RIPK3 (Figure 6B), or MLKL (Figure 6C), nor does it directly inhibit the kinase activity of these proteins.

### 3.7 HSP90AA1 is the core target of the PPI network

**FIGURE 7:**
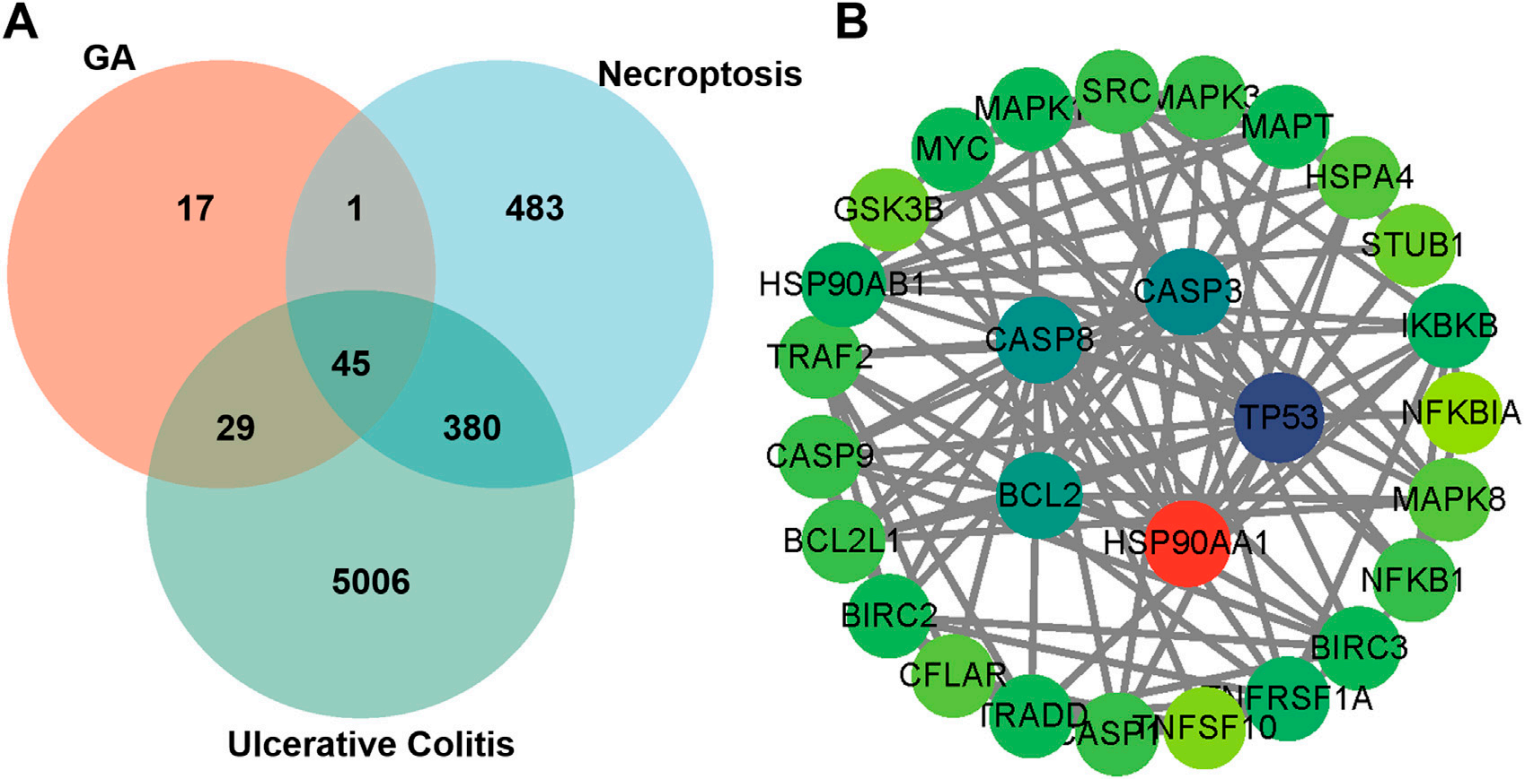
HSP90AA1 is the central of the GA PPI network. **(A)** Venn diagrams illustrate the 45 intersecting targets among GA, UC, and necroptosis. **(B)** The PPI network map highlights the intersecting targets shared by GA, UC, and necroptosis. The circle size indicates the significance of each gene target, with larger circles representing more important targets.

To investigate the potential targets of GA in the treatment of UC via necroptosis, we conducted a comprehensive analysis using target gene databases for GA, necroptosis, and UC. Initially, we identified 45 target genes by intersecting the gene sets associated with GA, UC, and necroptosis (Figure 7A). These 45 key genes were subsequently analyzed using the STRING database, with the species parameter set to “*Homo sapiens*”, to predict core proteins. A confidence score of 0.9 (highest confidence) was used on all interactions in order to minimize false positives/negatives. The resulting data were imported into Cytoscape software (version 3.10.2) in “tsv” format to construct a PPI network diagram (Figure 7B). Among the identified targets, HSP90AA1 was the node with the highest degree. Thus, HSP90AA1 (degree = 13) might as a core target of GA in the treatment of UC through necroptosis.

### 3.8 Molecular docking and molecular dynamics simulation results of GA and HSP90

**FIGURE 8:**
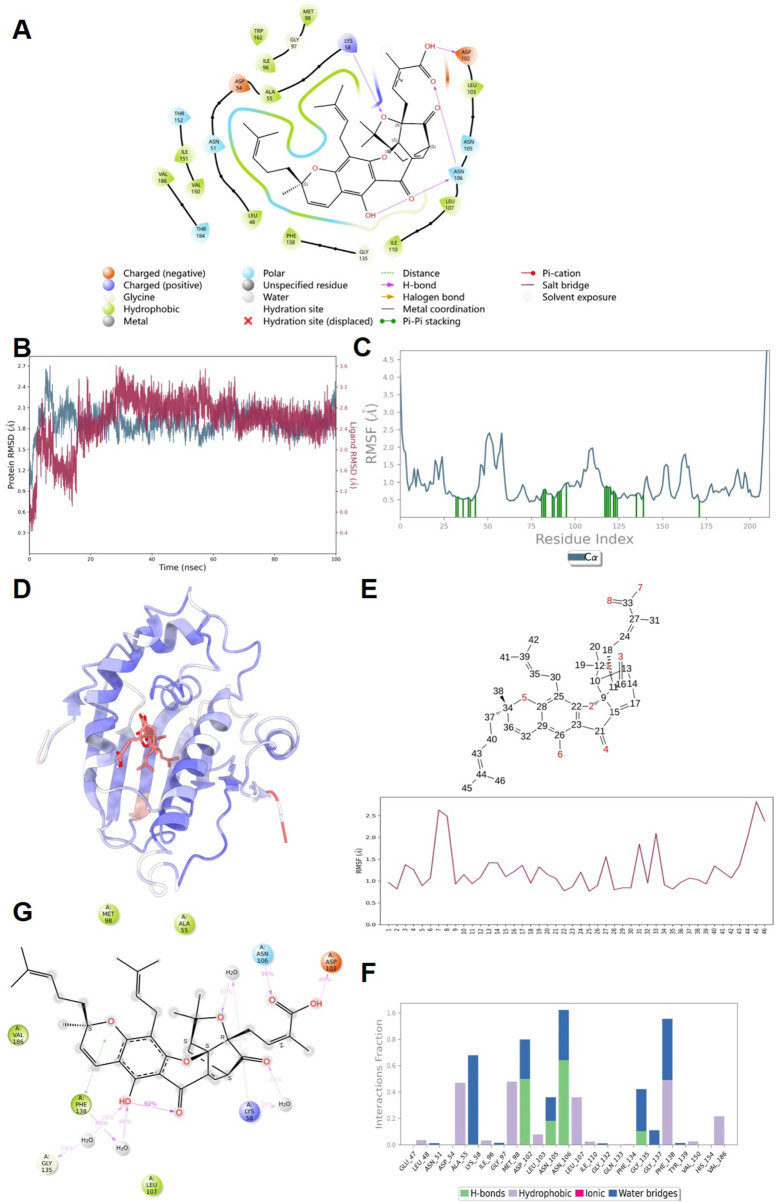
The results of molecular docking and molecular dynamics simulation. **(A)** Docking results of GA with HSP90 protein molecules. **(B)** RMSD; **(C,D)** Protein RMSF; **(E)** Ligand RMSF; **(F)** The connection between ligand and protein; **(G)** Ligand and protein Contacts (2 days-Summary).

Figure 8A presents the molecular docking results, demonstrating that GA can bind to HSP90. This binding is characterized by the formation of hydrogen bonds with the LYS58, ASP102, and ASN106 residues of HSP90. A 100 ns molecular dynamics simulation of the GA-HSP90 complex was conducted, followed by trajectory analysis. Initially, the RMSD values for both the protein and the small molecule were derived from the trajectory data. As shown in Figure 8B, the protein and GA achieved a stable conformation after 10 ns Therefore, subsequent analyses concentrated on the trajectories from 10 to 100 ns The RMSF values for the protein and the small molecule were also obtained, as illustrated in Figures 8C,D. Notably, the RMSF diagram for GA (Figure 8E) indicates that all amino acids of GA maintain a predominantly stable state. The interaction dynamics within the stability interval (10–100 ns) of the kinetic trajectory were examined, as shown in Figures 8F,G. Key amino acids involved in GA binding include ALA55, LYS58, MET98, ASP102, ASN106, GLY135, and PHE138, which primarily contribute through hydrogen bonding, water bridging, and hydrophobic interactions. The occupancy of interactions occurring within the 10–100 ns interval was quantified by calculating the ratio of frames displaying interactions to the total number of frames.

### 3.9 GA inhibits necroptosis pathway by targeting HSP90

**FIGURE 9:**
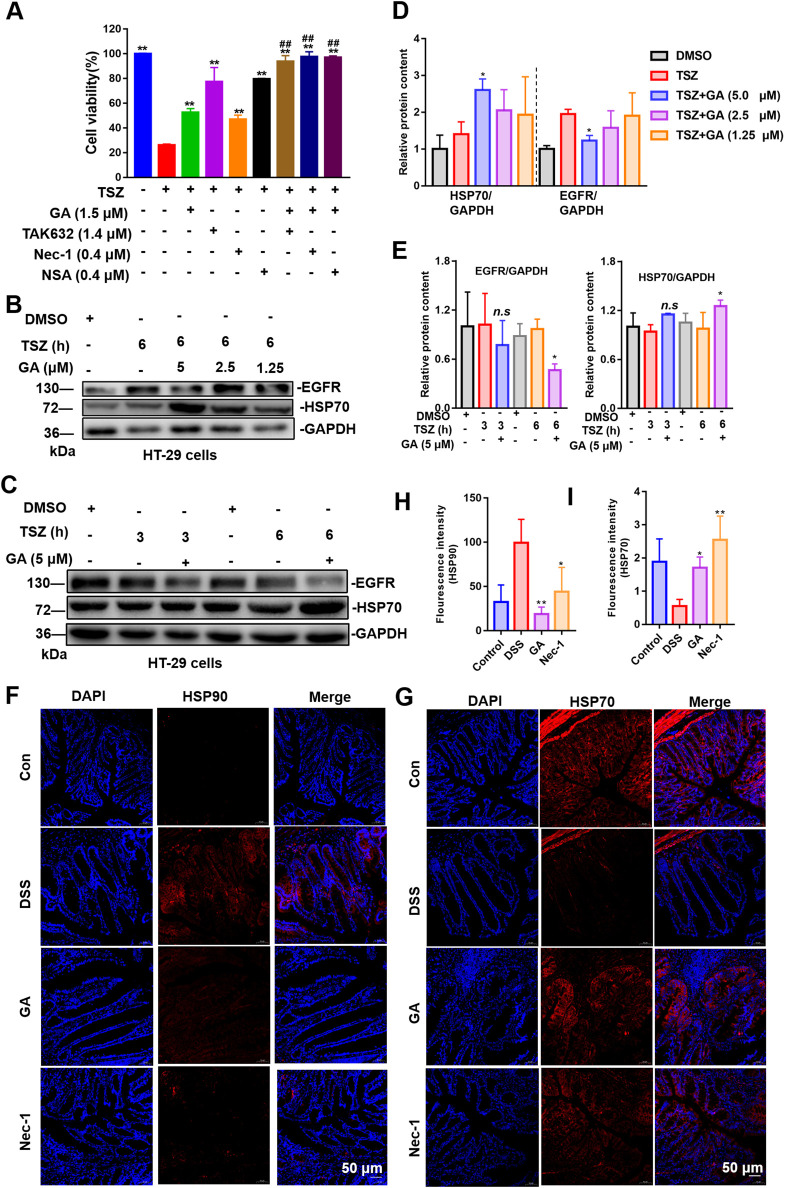
GA inhibits the necroptosis pathway by targeting HSP90. **(A)** HT-29 cells were treated with GA and other compounds, then stimulated with TSZ for 12 h to assess synergy effects. **(B)** HT-29 cells were exposed to different GA concentrations, followed by 6-h TSZ stimulation (n = 3). **(C)** HT-29 cells were pretreated with 5 μM GA for 30 min, then treated with TSZ for various duration (n = 3). **(D)** The quantitative analysis of the grayscale intensities of protein bands with different concentrations of GA for 6 h. **(E)** The quantitative analysis of the grayscale intensities of protein bands with different time. **(F)** The distribution and expression levels of HSP90 in colon tissues. **(G)** The distribution and expression levels of HSP70 in colon tissues. **(H)** Quantitative analysis of fluorescence intensity of HSP90. **(I)** Quantitative analysis of fluorescence intensity of HSP70.

Research has demonstrated RIPK1, RIPK3, and MLKL are regulated by HSP90 (Yang and He, 2016). The inhibition of HSP90 disrupts its interaction with RIPK1, leading to the degradation of RIPK1. Furthermore, HSP90 directly influences the stability and phosphorylation of RIPK3. Additionally, HSP90 is crucial for the oligomerization and membrane translocation of MLKL (Jacobsen et al., 2016). HSP90 inhibitors have been shown to ameliorate inflammation in ulcerative colitis by enhancing the secretion of the anti-inflammatory cytokine IL-10, increasing the number of regulatory T cells (Tregs), and attenuating inflammatory activation (Collins et al., 2013). Nevertheless, the precise role of HSP90 inhibition in the treatment of UC, particularly through the modulation of necroptosis, remains to be systematically explored. To investigate whether GA can inhibit the necroptosis signaling pathway via HSP90, we initially administered GA in combination with necroptosis inhibitors to assess cell viability. The results demonstrated that GA exhibited a synergistic effect when co-administered with Nec-1 (RIPK1 inhibitor), TAK632 (RIPK1/3 inhibitor), and NSA (MLKL inhibitor) (Figure 9A). This suggests that the target of GA is not RIPK1, RIPK3, and MLKL, and that its possible target is HSP90. Subsequently, we evaluated the impact of GA on HSP90 by measuring the levels of HSP70 and EGFR (the biomarkers of HSP90), which are indicative of HSP90 activity. Inhibition of HSP90 was associated with a significant upregulation of HSP70 and a significant downregulation of EGFR (Taipale et al., 2010). The findings indicated that GA could inhibit EGFR, a client protein of HSP90, and increase the expression level of HSP70 in a time-(Figures 9C,E) and dose-dependent (Figures 9B,D) manner. Finally, we assessed the expression levels of HSP70 and HSP90 in the colonic tissue of UC mice. In alignment with us *in vitro* findings, administration of GA led to an upregulation of HSP70 (Figures 9G,I) and a downregulation of HSP90 (Figures 9F,H). These results suggest that GA may modulate the necroptosis signaling pathway through the regulation of HSP90, thereby presenting a potential therapeutic strategy for the treatment of UC.

### 3.10 GA has no cardiac, hepatic or renal toxicity at effective doses

**FIGURE 10:**
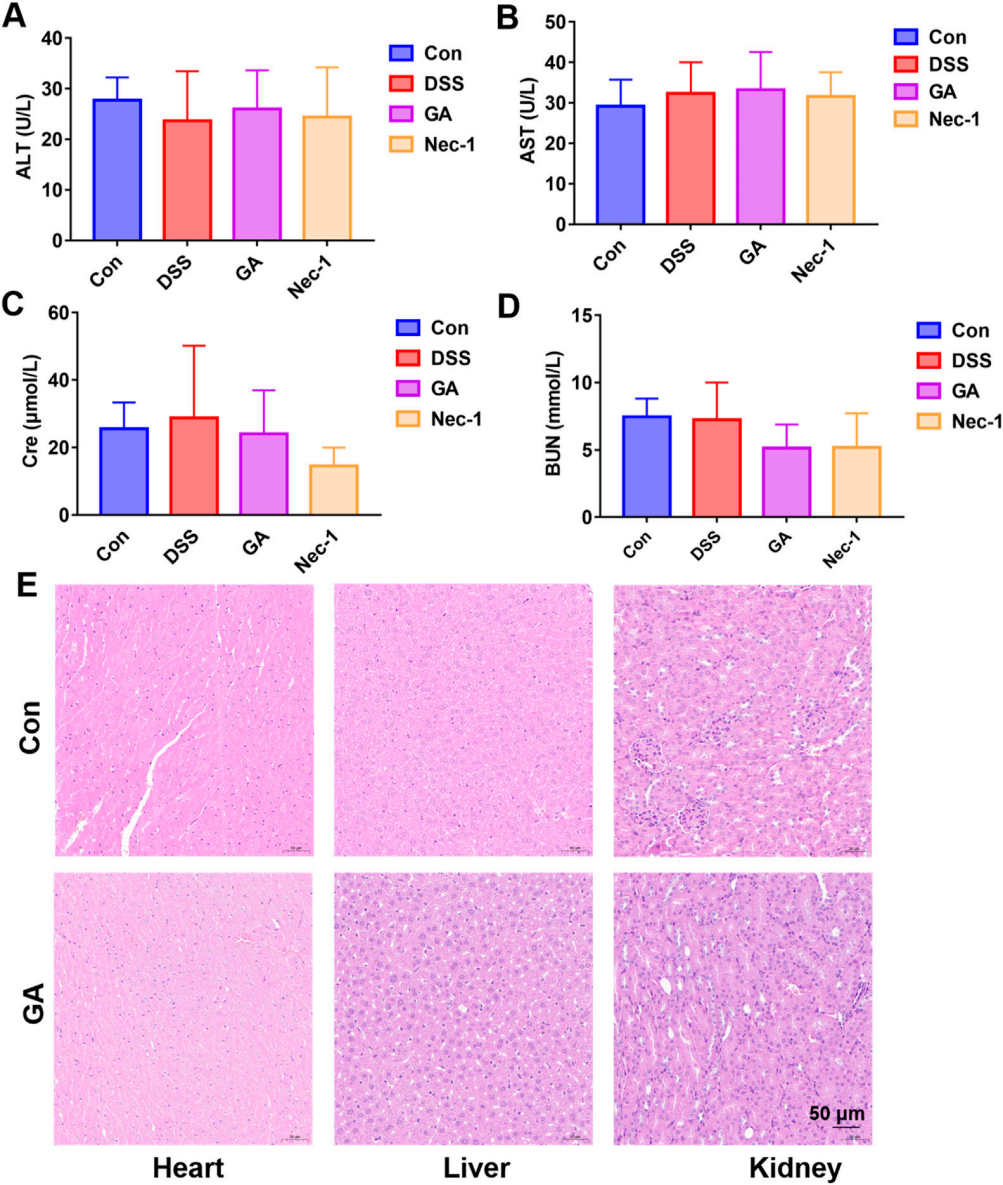
The toxicity of GA on cardiac, hepatic or renal in UC mice. **(A)** ALT; **(B)** AST **(C)** Cre; **(D)** BUN; **(E)** HE staining of heart, liver, and kidney sections.

Previous reports indicated that GA exhibited mild toxicity in the kidney and liver (Qi et al., 2008). Consequently, we assessed liver and kidney function and evaluated the extent of pathological damage in the liver and kidney in UC mice at the effective dose of GA used in this study. GA (5 mg/kg) does not influence the serum levels of ALT/AST (Figures 10A,B), nor does it impact the concentrations of BUN and CRE (Figures 10C,D). Furthermore, GA exhibited no pathological damage to the liver or kidneys in UC mice (Figure 10E). To further investigate the effects of GA on the pathology of heart, we also examined the cardiac injury of GA in UC mice (Figure 10E). GA exhibited no pathological damage to the heart in UC mice. Our experimental findings demonstrated that GA did not induce cardiac, hepatic, or renal toxicity at therapeutically effective doses for the treatment of UC.

## 4 Discussion

The aberrant death of IECs constitutes a primary factor contributing to the compromise of the intestinal barrier, further perpetuating a deleterious cycle with inflammatory responses. Necroptosis, a form of programmed cell death characterized by inflammatory properties, exacerbates the damage to the intestinal barrier when it occurs in IECs. Significant advancements have been made in the development of necroptosis inhibitors targeting critical components of the necroptosis signaling pathway. Numerous inhibitors have been identified and evaluated in preclinical trials; however, only a limited number have progressed to clinical testing and application. Since the discovery of the first necroptosis inhibitor, Nec-1 (Degterev et al., 2005), in 2005, it has demonstrated efficacy in preventing necrotic apoptosis-related diseases. However, the *in vivo* metabolic stability of Nec-1 is poor. GSK2982772 represents the inaugural RIPK1 inhibitor to undergo clinical trial evaluation (Harris et al., 2017). Clinical trial data indicate that GSK2982772 exhibits a favorable safety and tolerability profiles (Weisel et al., 2017). However, the therapeutic efficacy of GSK2982772 in the treatment of active UC (Weisel et al., 2021) has been found to be limited. SAR443060 is a selective, orally bioavailable, and permeable reversible inhibitor of RIPK1 (Vissers et al., 2022). Nevertheless, the clinical trial for SAR443060 has been discontinued due to long-term preclinical toxicity concerns.

Traditional Chinese medicine is distinguished by its efficacious therapeutic outcomes and minimal adverse effects. Natural products derived from traditional Chinese medicine offer several advantages, including abundant availability, high bioactivity, and low toxicity. Consequently, research into their pharmacological activities is gaining momentum. The process of necroptosis bears resemblance to the concept of “heat and toxicity” as articulated in traditional Chinese medicine theory. In this study, we developed a compound library aimed at heat clearance, detoxification, and detumescence, in accordance with traditional Chinese medicine principles. Concurrently, it investigated natural compounds for their anti-necroptosis activity and explored their potential molecular targets. Through systematic screening, it was determined that GA exhibits significant anti-necroptosis activities.

*Garcinia hanburyi* Hook. f., a traditional Chinese herbal medicine, is a dried resin secreted by Garcinia hanburyi trees after their trunks are cut. It has a sour and astringent taste and is known for its anti-inflammatory, detoxifying, and wound-healing properties. Historical texts such as the “Gangmu Shiyi” document its use in treating ulcers, bleeding, and various skin conditions (Zhao, 1963). GA is a major chemical component of *Garcinia hanburyi* Hook. f. Modern research has revealed its diverse pharmacological effects, encompassing anti-inflammatory, anti-tumor, and antibacterial activities. GA has shown promise in treating conditions such as lung injury, mastitis, arthritis, and myocardial infarction (Han et al., 2020). Its anti-inflammatory mechanisms may involve the inhibition of NF-κB, MAPK, and TrkA/Akt signaling pathways (Gao et al., 2021). However, its role and mechanism of action in UC have not been previously reported in the literature. In this study, GA was found to inhibit the phosphorylation of RIPK1, RIPK3, and MLKL in TSZ-induced HT29 cells in a dose-dependent manner. Furthermore, in a murine model of UC, GA ameliorated symptoms in a murine model of UC and attenuated necroptosis in IECs, thereby enhancing the integrity of the intestinal barrier and mitigating intestinal inflammation.

HSP90 is an ATP-dependent molecular chaperone that is essential for protein folding and maturation, operating through a chaperone cycle that incorporates various cochaperone proteins (Wang et al., 2022). The inhibition of HSP90 holds the potential to attenuate the necroptosis signaling pathway (Yang and He, 2016). These kinase proteins, RIPK1, RIPK3, and MLKL, have been identified as client proteins of HSP90. Dysfunctions in HSP90 can result in the degradation of these client proteins via the ubiquitin-proteasome pathway. Moreover, the inhibition of HSP90 can obstruct the formation of the necrosome, the phosphorylation of RIPK3, and the occurrence of necroptosis. In cases of non-RIPK1-dependent, polymerized RIPK3-induced necrosis, the function of HSP90 can be effectively disrupted. In 293T cells lacking RIPK3 expression, overexpression of HSP90 enhanced MLKL-induced cell death. The inhibition of HSP90 function could prevent the oligomerization and aberrant membrane localization of the mutant MLKL S345D (Jacobsen et al., 2016; Jacobsen and Silke, 2016). Thus, HSP90 is implicated in the activation of RIPK1, RIPK3, and MLKL in multiple ways. Simultaneously, the inhibition of HSP90 has been shown to ameliorate symptoms of UC. Research indicates that HSP90 inhibitors can attenuate the inflammatory response in UC by augmenting the secretion of the anti-inflammatory cytokine IL-10, increasing the population of regulatory T cells, and reducing the activation of the inflammasome (Collins et al., 2013; Shaaban et al., 2022). Therefore, the strategic targeting of HSP90 and the regulation of necroptosis may offer a novel therapeutic approach for the treatment of UC. In this study, the potential target genes associated with GA, UC, and necroptosis were intersected. The intersecting genes were subsequently ranked based on their degree values using Cytoscape software. Among these, HSP90AA1 emerged as the core target. *In vivo* and *in vitro* experiments have demonstrated that GA potentially inhibits necroptosis in IECs through the modulation of HSP90. Currently, many studies have shown that there are many pathways that can be associated with necrptosis. GA may exert its effects through other pathways, such as NF - κB or MAPK pathways. Further investigation is warranted to explore any additional, yet unidentified targets of GA.

**FIGURE 11:**
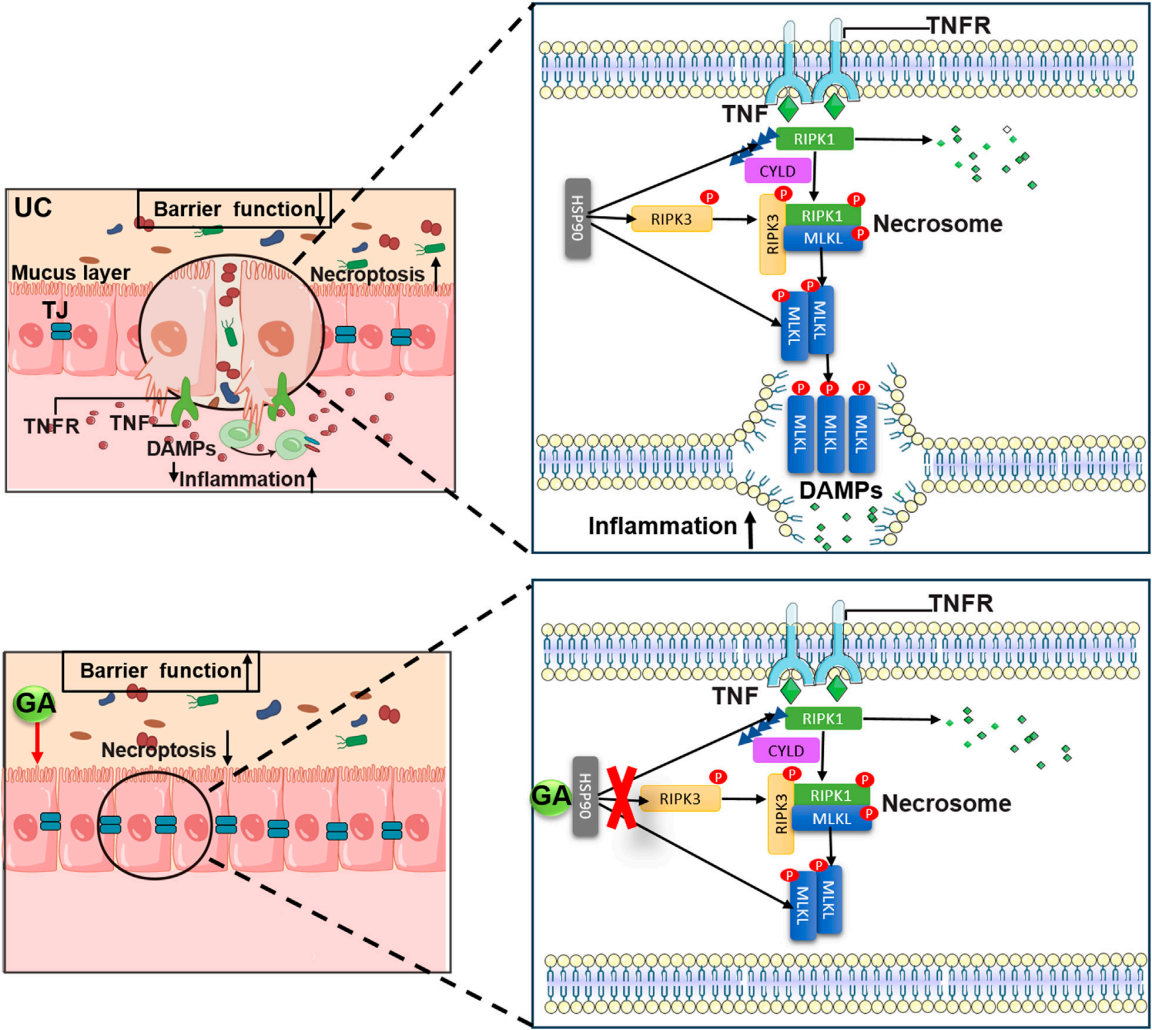
Schematic representation of proposed mechanism of GA on the protective effect of UC.

In conclusion, GA demonstrates the capacity to inhibit necroptosis in IECs and its anti-colitis effects may through the suppression of HSP90 expression. Our findings underscore the potential therapeutic application of HSP90 inhibition by GA in mitigating necroptosis and treating UC. The modulation of necroptosis in IECs via HSP90 inhibition presents a promising therapeutic strategy for UC and other conditions associated with necroptosis. The proposed mechanism is depicted in Figure 11.

## Data Availability

The original contributions presented in the study are included in the article/Supplementary Material, further inquiries can be directed to the corresponding authors.

## Abbreviations

IECs, intestinal epithelial cells; HSP90, heat shock protein 90; GA, gambogic acid; DSS, dextran sodium sulfate; UC, ulcerative colitis; TJ, tight junction; TNF-α, tumor necrosis factor-α; TNFR1, TNF receptor 1; RIPK1, receptor-interacting protein kinase1; RIPK3, receptor-interacting protein kinase 3; MLKL, mixed lineage kinase domain-like kinase; Nec-1, necrostain-1; Con, control; i. p., intraperitoneally; DAI, disease activity index; PAS, periodic acid-schiff; ELISA, enzyme-linked immune sorbent assay; PPI, protein-protein interaction; NCBI, national center for biotechnology information; JAMs, junctional adhesion molecules.
