# Supplementary material for: Gambogic acid targets HSP90 to alleviate DSS-induced colitis via inhibiting the necroptosis of intestinal epithelial cells
Source: Front Pharmacol. 2025 May 19;16:1586705. doi: 10.3389/fphar.2025.1586705 (PMC12127353; doi:10.3389/fphar.2025.1586705)

**<Supporting Information>**

1. **Supplement Figure 1**


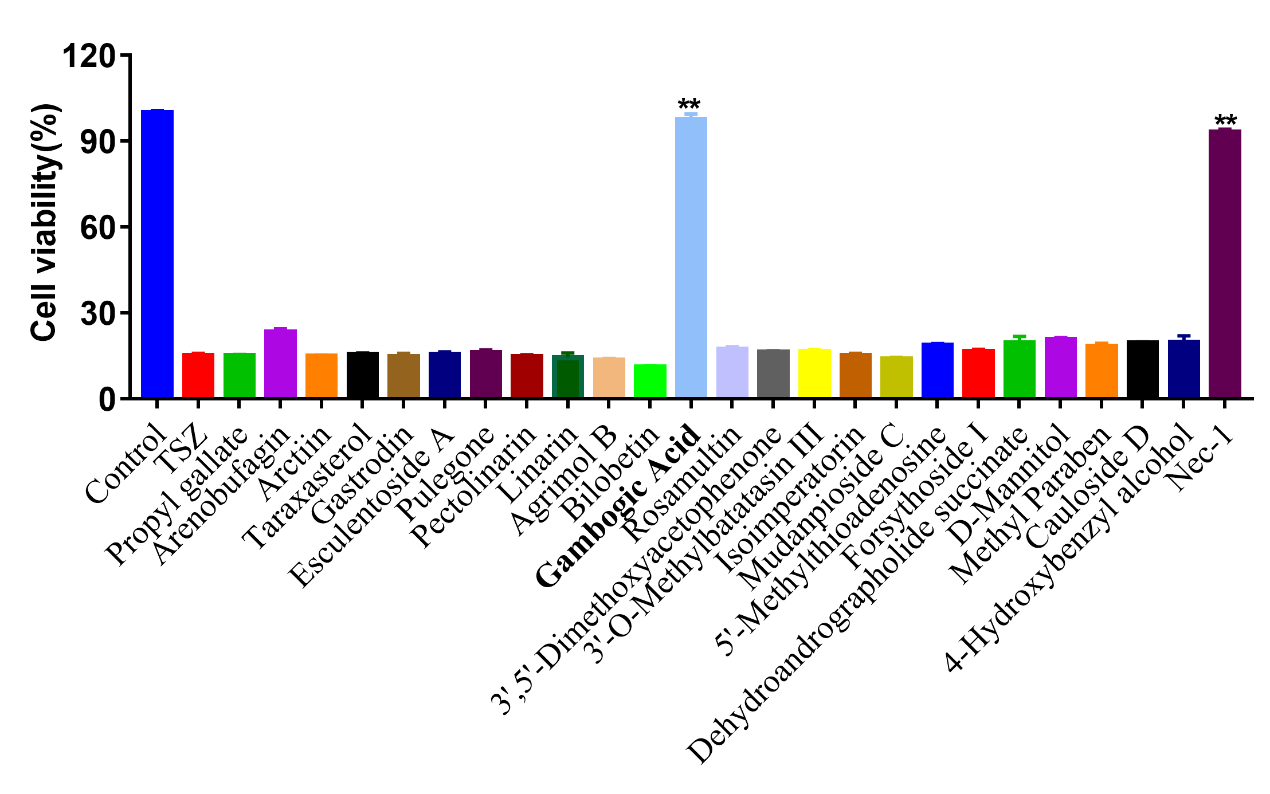


**Supplement Figure 1**

GA as a potential necroptosis inhibitor. Screening compounds for “clearing heat poison to dispel evil” and “anti-inflammatory”. The primary screening concentration of all compounds was 10 μM.

1. **The Full Bolts of Western Blots**


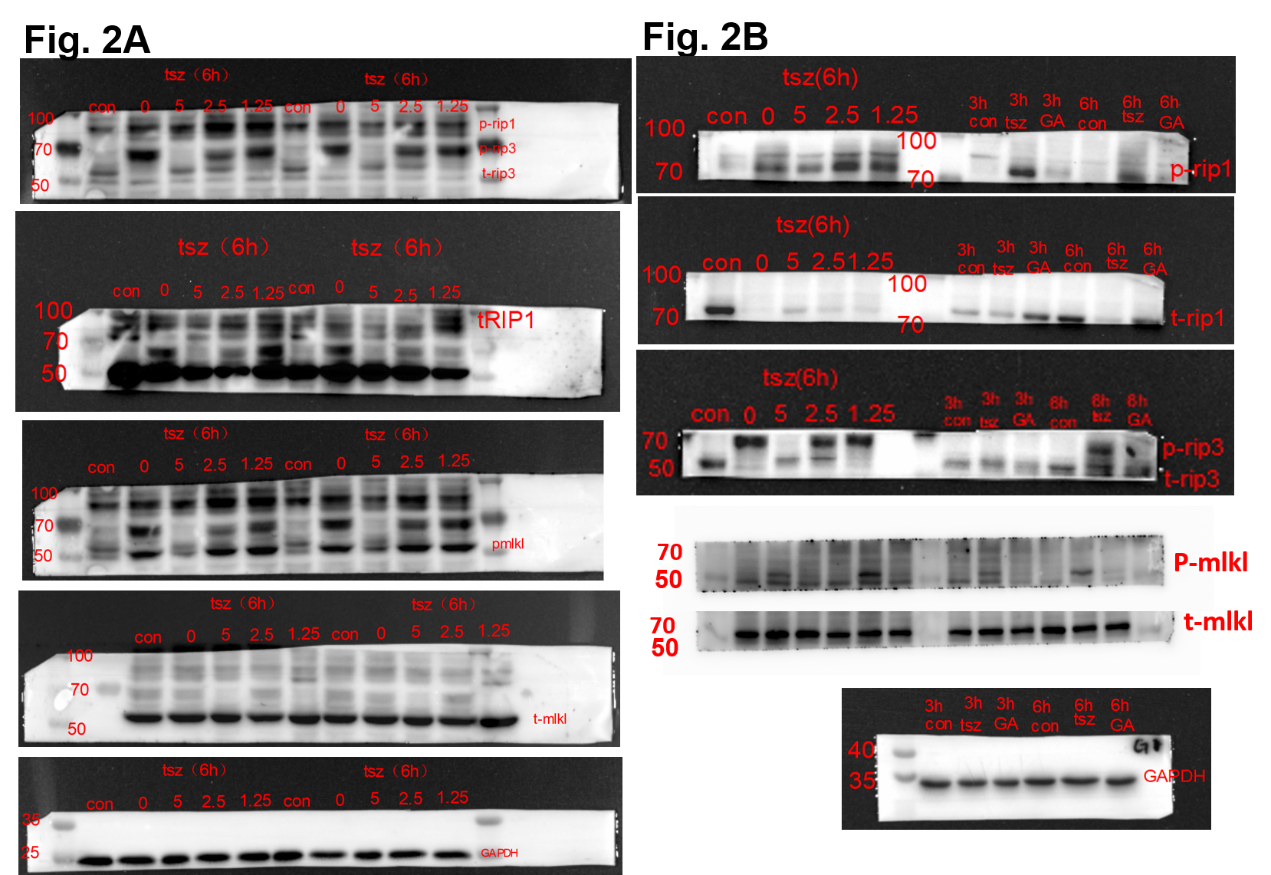


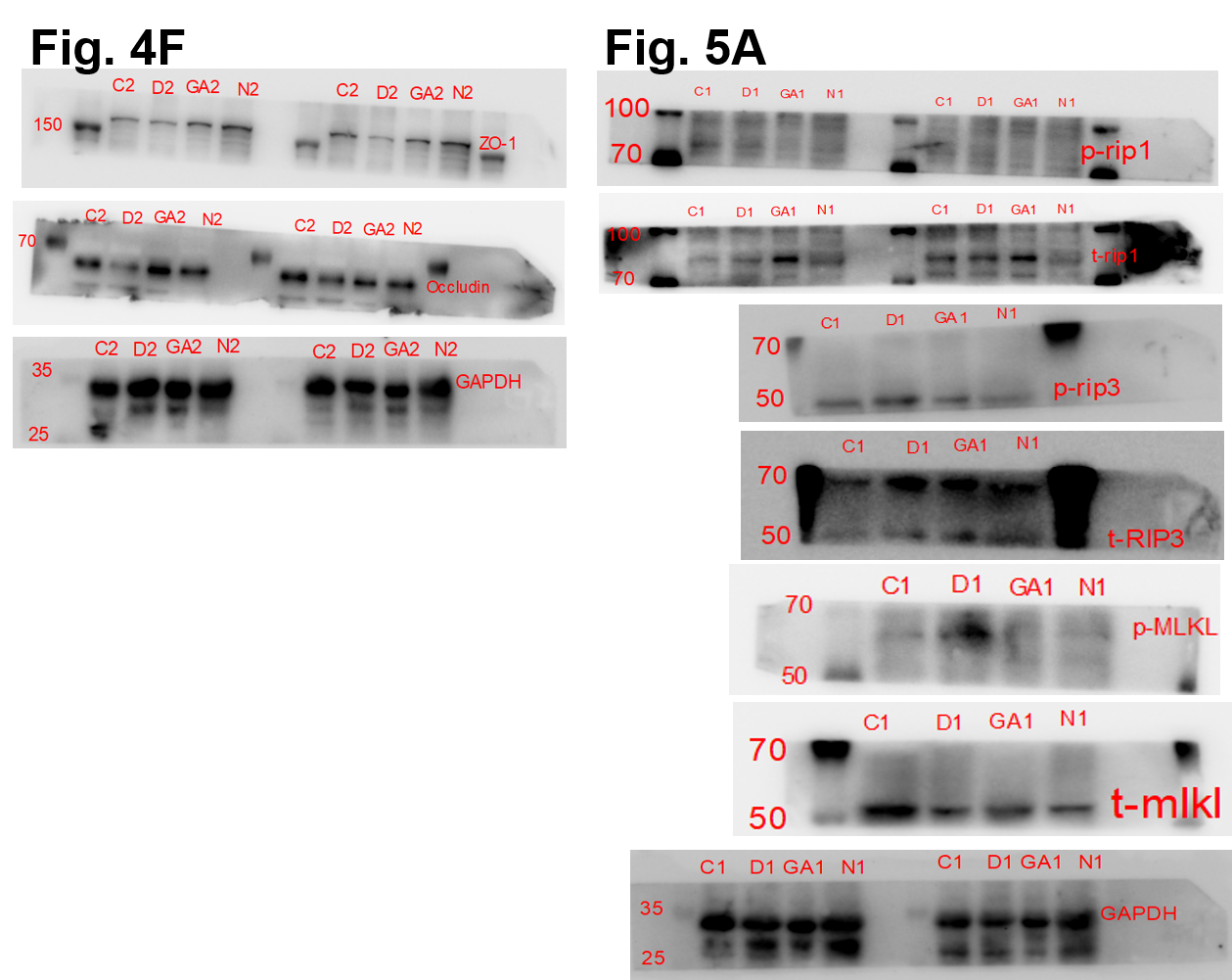


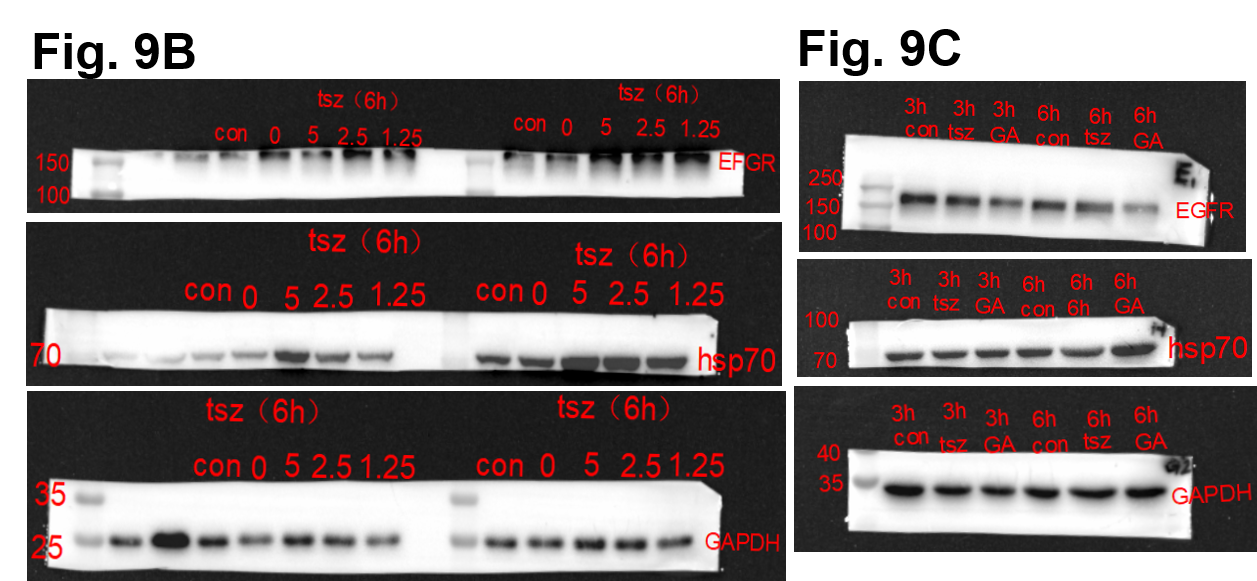

Supplement: Supplementary file 1 [file DataSheet1.docx]
